# Supplementary material for: Unassisted photoelectrochemical water splitting exceeding 7% solar-to-hydrogen conversion efficiency using photon recycling
Source: Nat Commun. 2016 Jun 21;7:11943. doi: 10.1038/ncomms11943 (PMC5476685; doi:10.1038/ncomms11943)
Supplement: Supplementary Information — Supplementary Figures 1-8, Supplementary Notes 1-2 and Supplementary References [file ncomms11943-s1.pdf]

Supplementary Figures

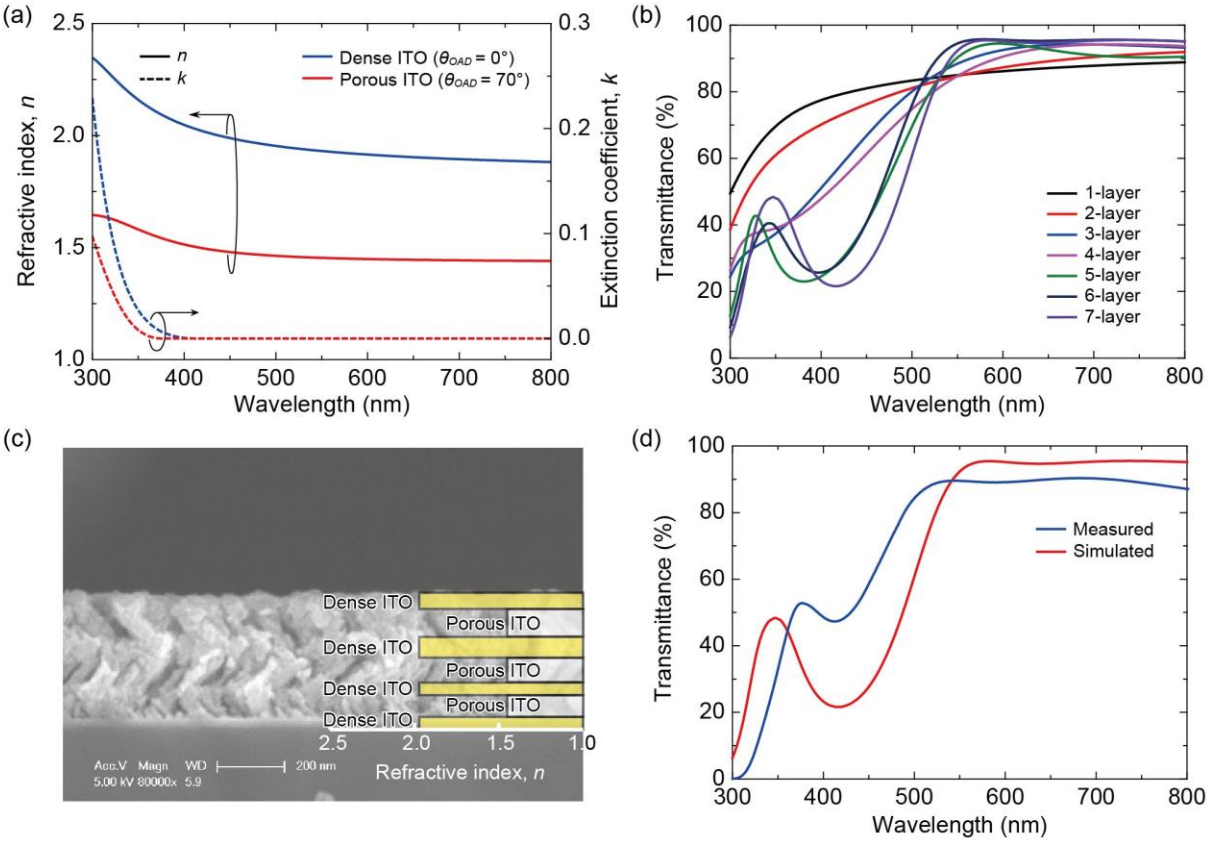

Supplementary Figure 1. Optical properties of ITO layers, and simulation and fabrication results of ITO cDBR. (a) The refractive index (solid lines) and the extinction coefficient (dashed lines) of dense ITO and porous ITO as a function of wavelength. (b) Simulated transmittance spectra of ITO cDBR upon varying the number of ITO layers (1-7 layers) (c) Cross-sectional SEM image of the fabricated cDBR with 7-layer ITO showing an indistinct interface between the layers. (d) Measured (blue line) and simulated (red line) transmittance spectra of the cDBR with 7-layer ITO. The absorption effect of the glass substrate at <360 nm was not taken into account in the GA simulation.

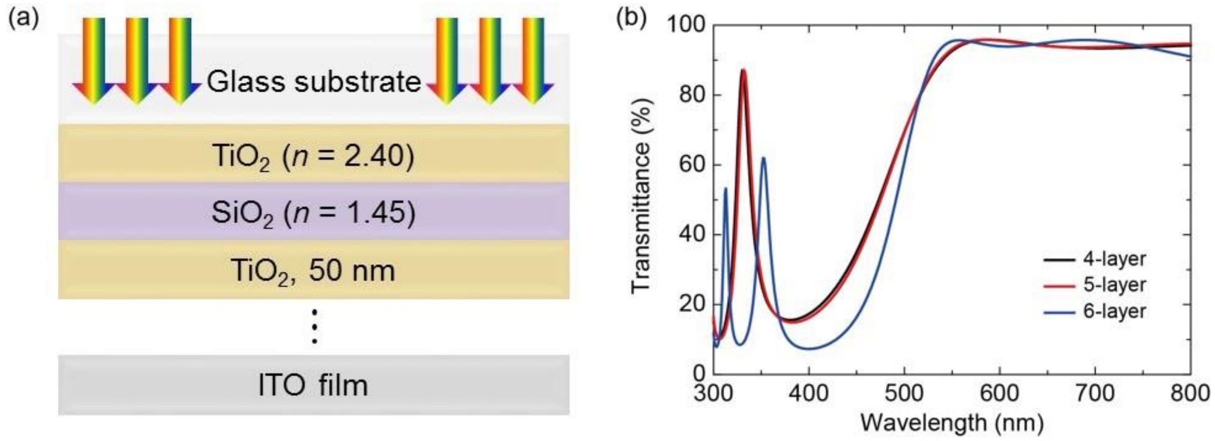

Supplementary Figure 2. Optimization of TiO_2/SiO_2 dielectric DBR framework. (a) Structure for the optimization of the TiO_2/SiO_2 stacks for the newly designed hybrid cDBR. (b) Simulated transmittance spectra of TiO_2/SiO_2 stacks from 4 to 6 layers.

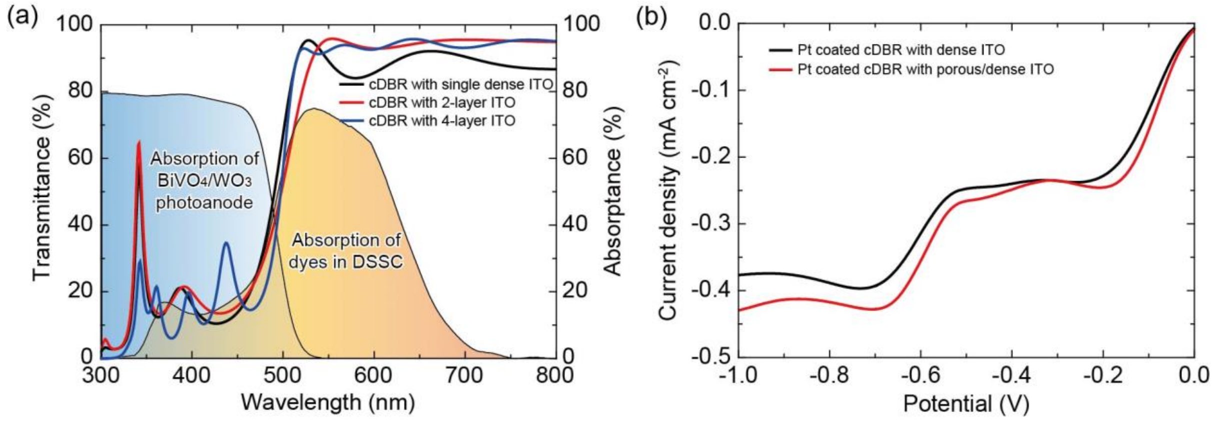

Supplementary Figure 3. Simulation results of optimized hybrid cDBR structures and cyclic voltammetry measurement. (a) Simulated transmittance of cDBR stacked with single dense ITO, 2-layer ITO and 4-layer ITO. (b) The reduction curves of cyclic voltammetry (CV) measurement for Pt coated cDBR with single dense ITO and porous/dense ITO, in which the 2-layer one showed higher cathodic peak current density. Pt deposition condition was following the process demonstrated in the methods part of DSSC fabrication with 20 times diluted electrolyte used for measurement.

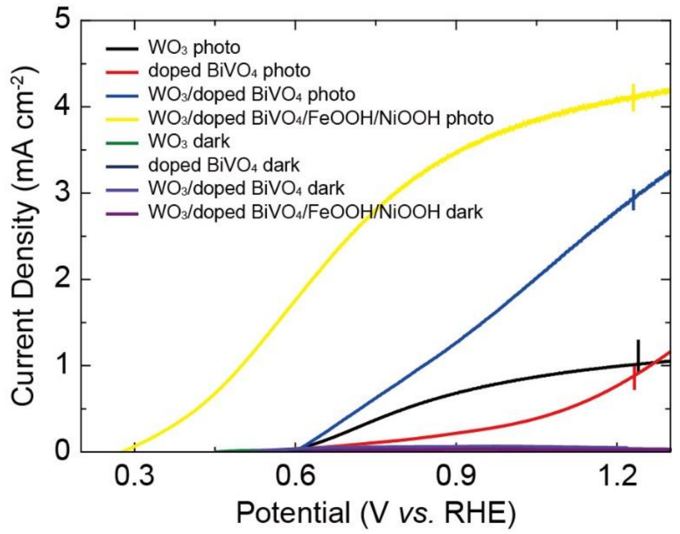

Supplementary Figure 4. PEC performances. PEC performance of photoanode under three-electrode system for both photo and dark current density, with the error bar marked at around 1.23V *vs.* RHE from the statistics of 5 samples for each condition.

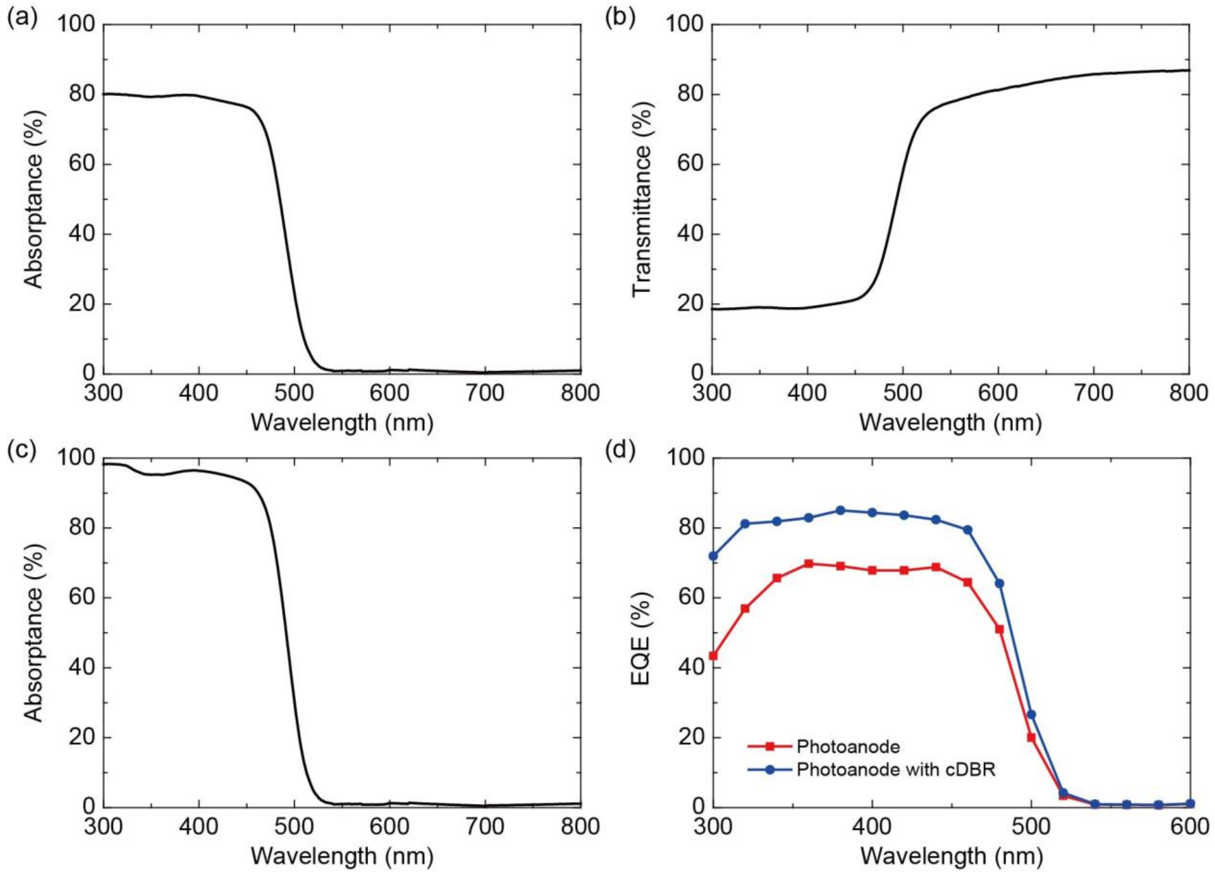

Supplementary Figure 5. Absorptance spectra of the photoanode. (a) before and (c) after the addition of the DBR stack. (b) Transmittance spectrum of the photoanode. (d) the experimental EQE results of photoanodes with and without cDBR.

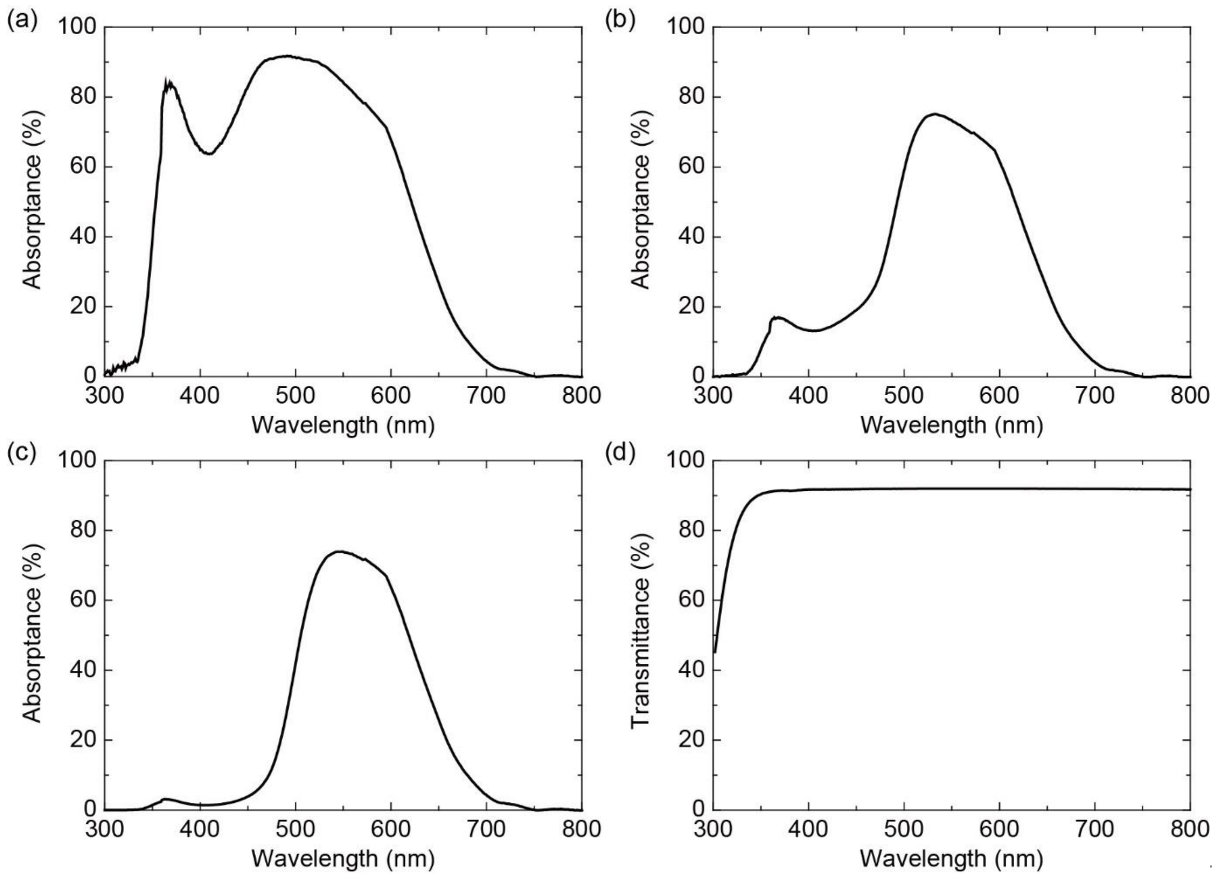

Supplementary Figure 6. Absorptance spectra of JK-306 dye-coated TiO_2 electrode used in the DSSC. (a) pristine measurements, (b) measured behind the $BiVO_4/WO_3$ photoanode, and (c) measured behind the $BiVO_4/WO_3$ photoanode and cDBR stack, (d) the transmittance of glass which is used for theoretical calculation for DSSC before addition of cDBR (for the case after addition of cDBR, T_{glass} is already included in T_{DBR})

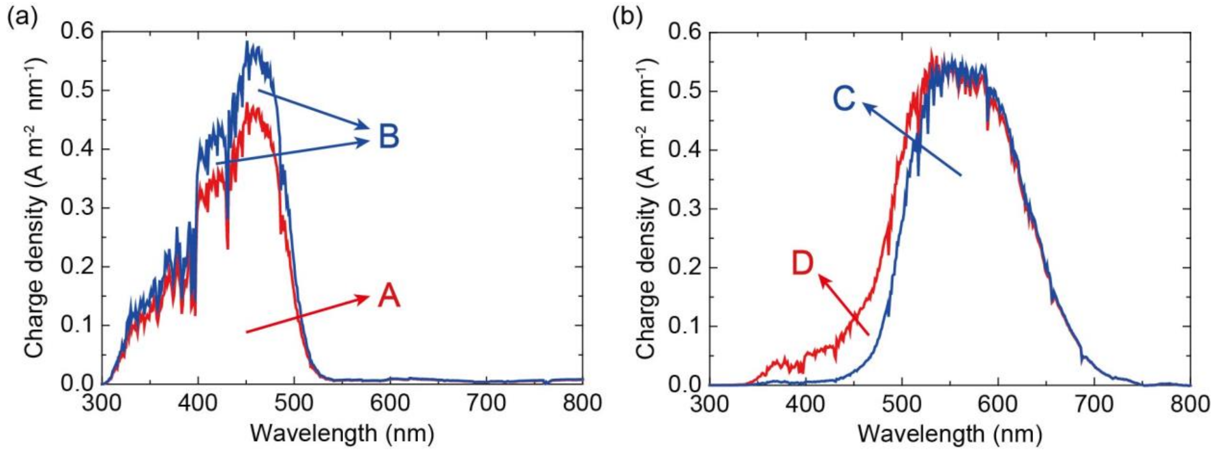

Supplementary Figure 7. Theoretical values obtained from the product of the solar spectrum and absorption data. (a) Charge density of the photoanode with (blue) and without (red) the DBR stack. The ratio of the integration areas for the two curves ($J_{abs,\ sum}$: $J_{abs,\ anode}$), corresponding to the areas (A+B) and A, is approximately 5:4. (b) Charge density of the DSSC without (red) and with (blue) the DBR stack. Similarly, the ratio of the area (C+D)/C, namely, $J_{abs,\ rear1}$: $J_{abs,\ rear2}$, is approximately 8:7. The charge density is obtained from absorption × photo flux (corresponding to 1sun 1.5G AM illumination)×e, where e is the charge of one electron, which is equal to 1.6×10^{-19} Coulomb.

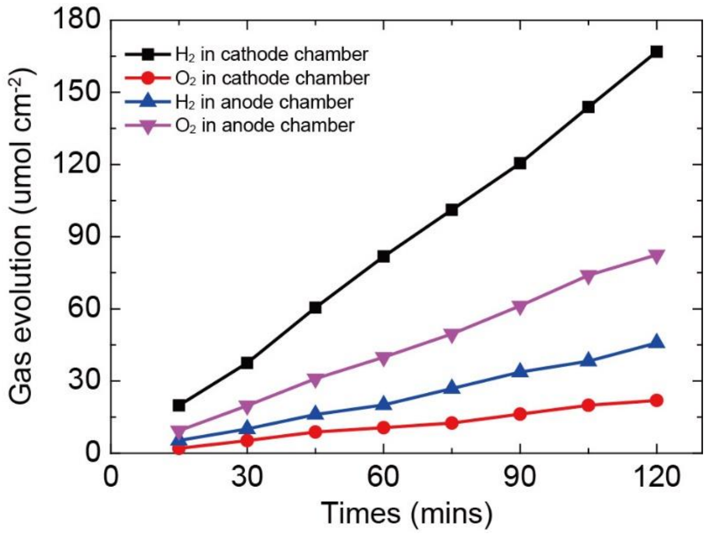

Supplementary Figure 8. Gas evolution. The gas evolved in two chambers and analysed based on a time interval of 15mins, for the tandem device with hybrid cDBR with 2-layer ITO. The calculation results show that at the time 120mins, there're around 11.6% O_2 in cathode chamber (DSSC and Pt part) detected, while there's 35.7% H_2 in anode chamber (photoanode part) detected, in mole percentage.

Supplementary Notes

Supplementary Note 1: GA optimization calculation and design of the hybrid conductive DBR

In the GA run by MATLAB, the transfer matrix method[1] was used for calculating the reflectance R and the transmittance T of each cDBR. Transfer matrix of each layer is:

$$\mathrm{M}(j) = \begin{bmatrix} m_{11} & m_{12} \\ m_{21} & m_{22} \end{bmatrix} = \begin{bmatrix} \cos\beta_j & -\frac{i}{p_j}\sin\beta_j \\ -ip_j\sin\beta_j & \cos\beta_j \end{bmatrix} \tag{1}$$

where $\beta_j = \frac{2\pi}{\lambda_0} n_j h_j \cos\theta_j$ (by refraction, $n_j\sin\theta_j = n_{j-1}\sin\theta_{j-1}$), $p_j = n_j\cos\theta_j$ (for TE – polarized light), $p_j = \frac{n_j}{\cos\theta_j}$ (for TM – polarized light), n_j, h_j, θ_j are complex refractive index, height, and incident angle of jth layer, respectively, and λ_0 is the wavelength of incident light. The complex refractive index including index of refraction (n) and extinction coefficient (k) was used to take the absorption of light by each layer into account. Then, the calculated reflection and transmission coefficients were:

$$r = \frac{(m_{11}+m_{12}p_l)p_1-(m_{21}+m_{22}p_l)}{(m_{11}+m_{12}p_l)p_1+(m_{21}+m_{22}p_l)}, \qquad R = |r|^2 \tag{2}$$

$$t = \frac{2p_1}{(m_{11}+m_{12}p_l)p_1+(m_{21}+m_{22}p_l)}, \qquad T = \frac{p_l}{p_1}|t|^2 \tag{3}$$

where '1' and 'l' denote the first and last layer of multilayer films. GA optimization calculations begin with the generation of a population of multilayer cDBR films with a fixed

number of layers whose thicknesses and compositions are randomly generated, followed by the evaluation of the FOM of each member, as described in the main text.

First, cDBR stacks using only ITO films were designed. Because ITO films with different porosities could have different refractive indices [2], this single material could allow the fabrication of the cDBR. For GA optimization, the measured refractive index as well as the extinction coefficient profiles of the dense (θ_{OAD} = 0°) and porous (θ_{OAD} = 70°) ITO layers fabricated by OAD were used (**Supplementary Fig. 1a**). cDBR structures with varying numbers of ITO layers (1-7 layers) were designed in the GA optimization, with the 7-layer specimen showing slightly superior optical properties (**Supplementary Fig. 1b**). However, the interface between the dense layer and the porous layer was not completely distinct when the designed 7-layer structure was fabricated by OAD using electron-beam evaporation (**Supplementary Fig. 1c**). This indistinct interface led to inconsistencies between the experimental and simulated transmittances [3], as shown in **Supplementary Fig. 1d**.

Therefore, we re-designed the hybrid cDBR structures composed of only a few conductive ITO layers stacked on a conventional dielectric DBR framework consisting of two different dielectric thin films with a high refractive index contrast. TiO_2 (n=2.40 at λ=550 nm) and SiO_2 (n=1.45 at λ=550 nm) thin films were used for the dielectric DBR stack located under the ITO film and were optimized by varying the number of alternating layers of TiO_2/SiO_2 thin films from 4 to 6 to compare their performances, as shown in **Supplementary Fig. 2**. The dielectric DBR with 6 layers of TiO_2 and SiO_2 showed the best result, showing the highest reflectance in the spectrum with a wavelength <500 nm and a high transmittance at a wavelength >500 nm. The hybrid cDBR structures were further optimized as composites of

dense and porous ITO stacks on the TiO_2/SiO_2 6-layer DBR framework. To preserve the electrical properties comparable to those of the ITO film, the dense ITO layer was used as the starting bottom layer of the ITO stacks in the GA optimization process because the bottom layer primarily determines the electrical conductivity of the hybrid cDBR structure. **Supplementary Fig. 3a** showed the calculated transmittance of the hybrid cDBR structures with single dense ITO top layer, and 2-layer, and 4-layer porous/dense (porous on top of dense) ITO stacks. The cDBR with 2-layer and 4-layer ITO showed higher FOM of 1.3186 and 1.3238, respectively, than that with single dense ITO layer (FOM = 1.3037), indicating that the optical performance of the cDBRs with 2- and 4-layer ITO stacks (with porous ITO on the top) was superior to that of the cDBR with optimized single dense ITO layer. In addition to the optical benefit, a porous layer was intentionally chosen as the topmost layer for experiments because a porous framework for Pt coating would be beneficial for a high performance of DSSC enabled by a higher surface area and electrocatalytic activity than the planar one [4] (**Supplementary Fig. 3b**). As a result, the hybrid cDBRs with 2-layer and 4-layer ITO stacks were fabricated and used in this work, as described in the main text.

Supplementary Note 2: Theoretical studies of the optical function of the hybrid cDBR

Theoretical studies were conducted to investigate the optical function of the hybrid cDBR used in the tandem system. This study is mainly based on the significant linear relationship among light absorption, reflection and transmission using relevant calculations.

1. Photoanode

In the supplementary note 1, the absorption of cDBR is considered to get more accurate theoretical data and reduce the variances between the simulated and experimental curves. In this part, to make the expression more clearly the cDBR is regarded as an ideal dielectric light filter, which means the light that cannot be transmitted is completely reflected. This assumption will not affect the final estimation of the integration ratios for photoanode and DSSC as shown below. Therefore, in this work, we name the experimental data of the transmittance curve as T_{DBR} (T_{DBR} has included the transmittance of FTO glass since DBR is fabricated on the back of FTO/glass and we consider them together in theoretical calculation), and the reflected light can be expressed as $1 - T_{\mathrm{DBR}}$. The absorption and transmittance of the photoanode are shown in **Supplementary Figs 5a** and **b** and given the names A_{anode} and T_{anode}, respectively. The reflectance of the photoanode can then be expressed as $1 - A_{\mathrm{anode}} - T_{\mathrm{anode}}$. When light illuminates from the front photoanode to the DBR and then to the DSSC, as illustrated in the schematic in **Fig. 1,** the T_{anode} component of the solar spectrum will penetrate photoanode and reach DBR. Among these spectra, the ratio $(1 - T_{\mathrm{DBR}})$ will be reflected back by the DBR, which is $T_{\mathrm{anode}} \times (1 - T_{\mathrm{DBR}})$. Those spectra will reach the photoanode again and be divided into three parts, namely, the components that are absorbed by the photoanode, reflected by the photoanode, and penetrate the photoanode.

Given that the absorption of the photoanode is A_{anode}, the secondary absorption of the photoanode is $T_{\text{anode}} \times (1 - T_{\text{DBR}}) \times A_{\text{anode}}$. The light reflected by the photoanode, $T_{\text{anode}} \times (1 - T_{\text{DBR}}) \times (1 - A_{\text{anode}} - T_{\text{anode}})$, will reach the DBR again and be reflected again; subsequently, this reflected light will reach the photoanode and cause the tertiary absorption of the photoanode, $T_{\text{anode}} \times (1 - T_{\text{DBR}}) \times (1 - A_{\text{anode}} - T_{\text{anode}}) \times A_{\text{anode}}$. By the same token, quaternary absorption, $T_{\text{anode}} \times (1 - T_{\text{DBR}})^2 \times (1 - A_{\text{anode}} - T_{\text{anode}})^2 \times A_{\text{anode}}$, will also occur. Considering an ideal case, the absorption of the photoanode after the addition of the DBR, A_{sum} is

$$\begin{aligned} A_{\text{sum}} = {} & A_{\text{anode}} + T_{\text{anode}} \times (1 - T_{\text{DBR}}) \times A_{\text{anode}} \\ & + T_{\text{anode}} \times (1 - T_{\text{DBR}}) \times (1 - A_{\text{anode}} - T_{\text{anode}}) \times A_{\text{anode}} + \cdots \\ & + T_{\text{anode}} \times (1 - T_{\text{DBR}})^{\text{n}} \times (1 - A_{\text{anode}} - T_{\text{anode}})^{\text{n}} \times A_{\text{anode}} \\ = {} & A_{\text{anode}} + T_{\text{anode}} \times (1 - T_{\text{DBR}}) \times A_{\text{anode}} + T_{\text{anode}} \times A_{\text{anode}} \\ & \times \frac{[(1- T_{\text{DBR}})\times (1- A_{\text{anode}}- T_{\text{anode}})]-[(1- T_{\text{DBR}})\times (1- A_{\text{anode}}- T_{\text{anode}})]^{\text{n}+1}}{1-[(1- T_{\text{DBR}})\times (1- A_{\text{anode}}- T_{\text{anode}})]} \end{aligned} \quad (4)$$

When the value of n trends towards positive infinity, the term $[(1 - T_{\text{DBR}}) \times (1 - A_{\text{anode}} - T_{\text{anode}})]^{\text{n}+1}$ in Supplementary Equation (4) will be zero. Thus the curve of A_{sum}, shown in **Supplementary Fig. 5c,** could be drawn based on the data in **Fig. 2** and **Supplementary Figs 5a** and **b**.

Considering the intrinsic property of the photoanode, the charge separation efficiency η_{sep} and charge transfer efficiency η_{tran} are not affected by the addition of the DBR stack. Therefore, the PEC performance of the photoanode with (J_{sum}) and without (J_{anode}) the DBR is dependent on J_{abs}, which is the theoretical maximum value after considering the light harvesting ability as shown in Supplementary Equation (5),

$$\frac{J_{\mathrm{sum}}}{J_{\mathrm{anode}}} = \frac{J_{\mathrm{abs,sum}} \times \eta_{\mathrm{sep}} \times \eta_{\mathrm{tran}}}{J_{\mathrm{abs,anode}} \times \eta_{\mathrm{sep}} \times \eta_{\mathrm{tran}}} = \frac{J_{\mathrm{abs,sum}}}{J_{\mathrm{abs,anode}}} \quad (5)$$

J_{abs} can be obtained by the integration of the product of the photo flux (corresponding to the intensity of 1.5G AM) and the absorption efficiency (**Supplementary Fig. 5a** A_{anode}, and **Supplementary Fig. 5c** A_{sum}).

The charge density curves obtained from the absorption efficiency curves multiplied by the photo flux for the photoanode with/without DBR are shown in **Supplementary Fig. 7a**. The ratio of the two areas, (A+B)/A, which corresponds to the values of $\frac{J_{\mathrm{abs,sum}}}{J_{\mathrm{abs,anode}}}$ and $\frac{J_{\mathrm{sum}}}{J_{\mathrm{anode}}}$, is approximately 5:4. This value is consistent with the value obtained in the PEC experiments with the photoanode shown in **Fig. 3a**. Besides, in order to verify the spectral response with/without cDBR and understand the function of cDBR to photoanode experimentally, the external quantum efficiency (EQE) has been checked and shown in **Supplementary Fig. 5d.** The results basically agrees well with the theoretical analyses.

2. DSSC

In the same way, the absorption curve of Dye JK-306 for the *in situ* measurement can be named as A_{DSSC} (**Supplementary Fig. 6a**). When the photoanode is placed in front of the DSSC, only the light penetrating through the photoanode and glass can be absorbed, which is named as A_{rear1} and calculated as $T_{\mathrm{anode}} \times T_{\mathrm{glass}} \times A_{\mathrm{DSSC}}$ (**Supplementary Fig. 6b**). Note that since T_{DBR} has included the transmittance of glass, while the data of T_{anode} has excluded the glass, we have to re-multiply the T_{glass} here. The T_{anode} and T_{glass} used for the calculation are shown in **Supplementary Fig. 5b** and **Supplementary Fig. 6d**, respectively.

Then, when the photoanode and DBR are both placed in front of the DSSC, the light penetrating through the photoanode T_{anode} will reach the DBR first, where it will be divided into two parts: one penetrates the DBR and reaches the DSSC and another is reflected by the DBR and reaches the photoanode again. Therefore, in this case, the primary absorption of the DSSC will be $T_{\text{anode}} \times T_{\text{DBR}} \times A_{\text{DSSC}}$. Some of the light that reaches the photoanode will be reflected, which is $T_{\text{anode}} \times (1 - T_{\text{DBR}}) \times (1 - A_{\text{anode}} - T_{\text{anode}})$. This part of the light reaches the DBR and DSSC and causes the secondary absorption of the DSSC, $T_{\text{anode}} \times (1 - T_{\text{DBR}}) \times (1 - A_{\text{anode}} - T_{\text{anode}}) \times T_{\text{DBR}} \times A_{\text{DSSC}}$. By the same token, the tertiary absorption of the DSSC is $T_{\text{anode}} \times (1 - T_{\text{DBR}})^2 \times (1 - A_{\text{anode}} - T_{\text{anode}})^2 \times T_{\text{DBR}} \times A_{\text{DSSC}}$. Considering an ideal case, the absorption of the DSSC after the addition of both the photoanode and DBR, A_{rear2} is,

$$\begin{aligned} A_{\text{rear2}} &= T_{\text{anode}} \times T_{\text{DBR}} \times A_{\text{DSSC}} + T_{\text{anode}} \times (1 - T_{\text{DBR}}) \times (1 - A_{\text{anode}} - T_{\text{anode}}) \\ &\quad \times T_{\text{DBR}} \times A_{\text{DSSC}} + \cdots \\ &\quad + T_{\text{anode}} \times (1 - T_{\text{DBR}})^{\text{n}} \times (1 - A_{\text{anode}} - T_{\text{anode}})^{\text{n}} \times T_{\text{DBR}} \times A_{\text{DSSC}} \\ &= T_{\text{anode}} \times T_{\text{DBR}} \times A_{\text{DSSC}} + T_{\text{anode}} \times T_{\text{DBR}} \times A_{\text{DSSC}} \\ &\quad \times \frac{[(1- T_{\text{DBR}})\times (1- A_{\text{anode}}- T_{\text{anode}})]-[(1- T_{\text{DBR}})\times (1- A_{\text{anode}}- T_{\text{anode}})]^{\text{n}+1}}{1-[(1- T_{\text{DBR}})\times (1- A_{\text{anode}}- T_{\text{anode}})]} \end{aligned} \tag{6}$$

When the value of n trends towards positive infinity, the term $[(1 - T_{\text{DBR}}) \times (1 - A_{\text{anode}} - T_{\text{anode}})]^{\text{n}+1}$ in Supplementary Equation (6) will be zero. Thus, the curve of A_{rear2}, shown in **Supplementary Fig. 6c**, could be obtained based on the data in **Supplementary Figs 5a** and **b**, **Supplementary Figs 6a** and **d**.

Similar to the photoanode section, the performance of the DSSCs behind the photoanode (J_{rear1}) and behind the photoanode/DBR (J_{rear2}) is dependent on their respective J_{abs}, namely,

$$\frac{J_{\text{rear1}}}{J_{\text{rear2}}} = \frac{J_{\text{abs,rear1}}}{J_{\text{abs,rear2}}} \quad (7)$$

J_{abs} can be obtained by the integration of the product of the photo flux and the absorption efficiency (**Supplementary Fig. 6b** A_{rear1}, and **Supplementary Fig. 6c** A_{rear2}).

The charge density curves obtained from the absorption efficiency curves multiplied by the photo flux for the two varieties of DSSC performance are shown as **Supplementary Fig. 7b**. The ratio of the two areas, (C+D)/C, which corresponds to the value of J_{rear1}:J_{rear2}, is approximately 8:7. This value is consistent with the experimental value obtained for the DSSC shown in **Fig. 3a.**

The theoretical analyses presented above verified the correction of the experimental data obtained in **Fig. 3a** and demonstrated the authentic function of the DBR, which could be used in the tandem system on the basis of the optical theories.

Supplementary References

[1] Born, M. & Wolf, E. *Principles of Optics: Electromagnetic Theory of Propagation, Interference and Diffraction of Light* 7th expanded edn (Cambridge Univ. Press, 1999)

[2] Schubert, M. F., Xi, J.-Q., Kim, J. K. & Schubert, E. F. Distributed Bragg reflector consisting of high- and low-refractive-index thin film layers made of the same material. *Appl. Phys. Lett.* **90**, 141115 (2007).

[3] Oh, S. J. *et al*. Enhanced phosphor conversion efficiency of GaN-based white light-emitting diodes having dichroic-filtering contacts. *J. Mater. Chem. C* **1**, 5733–5740 (2013).

[4] Bao, C. *et al*. The maximum limiting performance improved counter electrode based on a porous fluorine doped tin oxide conductive framework for dye-sensitized solar cells. *Nanoscale* **5**, 4951–4957 (2013)
